# Effectiveness of Robotic Stereotactic Radiotherapy in Patients Undergoing Re-irradiation: A Review

**DOI:** 10.7759/cureus.43500

**Published:** 2023-08-15

**Authors:** Ahamed Badusha Mohamed Yoosuf, Salem Alshehri, Mohd Zahri Abdul Aziz, Syahir Mansor, Gokula Kumar Appalanaido, Mamdouh Alqathami

**Affiliations:** 1 Oncology, King Abdullah International Medical Research Center, Riyadh, SAU; 2 Oncology/Radiation Oncology, King Abdulaziz Medical City, Riyadh, SAU; 3 Radiation Oncology, King Abdulaziz Medical City, Riyadh, SAU; 4 Advanced Management of Liver Malignancies Program, Universiti Sains Malaysia, Advanced Medical and Dental Institute, Penang, MYS; 5 Nuclear Medicine Unit, Pusat Perubatan Universiti Sains Malaysia, Advanced Medical and Dental Institute, Penang, MYS; 6 Radiotherapy Unit, Pusat Perubatan Universiti Sains Malaysia, Advanced Medical and Dental Institute, Penang, MYS; 7 Department of Oncology, Ministry of National Guard, Health Affairs, Riyadh, SAU; 8 Clinical Research, King Abdullah International Medical Research Center, Riyadh, SAU; 9 Radiological Sciences, King Saud Bin Abdulaziz University for Health Sciences, Riyadh, SAU

**Keywords:** stereotactic radiosurgery (srs), stereotactic radiosurgery, local control, overall survival (os), recurrence loco regional, re-irradiation, stereotactic body radiotherapy (sbrt), stereotactic ablative body radiotherapy, robotic stereotactic radiotherapy

## Abstract

Stereotactic ablative radiotherapy (SABR) is a possible treatment option for patients who develop recurrence within or at the edge of a previously irradiated volume. Robotic stereotactic radiotherapy is the result of technological advances in robotic precision, real-time imaging, non-invasive, highly customizable treatment plan, and delivery with sub-millimeter accuracy. This article reviews the radiobiologic, technical, and clinical aspects of robotic-based SABR re-irradiation for various anatomical sites. An extensive literature search was performed to identify articles on the utilization of robotic stereotactic radiotherapy for patients undergoing re-irradiation. The reported prescription dose and fractionation data along with outcomes such as overall survival, local control rates, and toxicities were qualitatively reviewed. The findings consistently indicate that re-irradiation using robotic SABR provides encouraging survival rates with minimal toxicity in the clinical setting of various anatomical sites delivered using locally non-invasive means where other treatment options are scarce.

## Introduction and background

The goal of radiation therapy (RT) is to deliver a therapeutically effective dose to the target while reducing damage to nearby normal tissues. In the last few decades, a variety of cutting-edge treatments have emerged in the field of radiation oncology like stereotactic ablative radiotherapy (SABR), delivery efficiency using flattening filter-free beams, real-time imaging, and CyberKnife-based robotic radiotherapy. There have been significant advancements in cancer detection, staging, and management, resulting in improved disease outcomes and survival rates [1]. Compared to perhaps three or four decades, patients receiving appropriate care are expected to live longer and have a higher quality of life. The issue of localized disease recurrence in an otherwise healthy patient necessitating extra modes of treatment for local control and palliation has come into focus as a result of the increased cure rate and survival.

Re-irradiation is a treatment option for recurrent or persistent tumors after primary RT. However, this poses a challenge because of the cumulative radiation dose and possible radiation toxicity to surrounding normal tissues. The extent of normal tissue regeneration can be affected by a variety of factors, including the presence of residual tissue injury, the time interval between two courses of radiation, the volume of tissue needed for re-irradiation, the fractionation schedule used in the prior course, the higher dose per fraction, and the late effect. The severity of the disease and, consequently, the likelihood of survival, the use of chemotherapy and/or surgery, and the RT technique being applied, all have an impact on tissue tolerance.

The increased use of innovative methods and technology, particularly in radiotherapy, has led to better normal tissue sparing, which has improved the quality of life. Owing to the ability of newer technologies to spare important and slow-reacting tissues, many clinicians are now considering re-irradiation. Examples of these technologies include robotic stereotactic radiosurgery (SRS)/stereotactic radiotherapy (SRT), intensity-modulated radiotherapy (IMRT), image-guided radiotherapy (IGRT), and brachytherapy [1]. The capacity to apply these techniques to calculate the doses to different normal tissues within the irradiated volume is the single most crucial factor in the management of re-irradiation. Robotic SRT, such as CyberKnife (Accuray, Sunnyvale, CA), has shown promise in delivering high precision and conformal dose distribution, potentially minimizing the risk of toxicity while maximizing tumor control.

The review focuses on patients with locally recurrent tumors previously irradiated with conventional external beam radiation therapy (EBRT) or brachytherapy and metastatic tumors from a variety of primary tumors with or without nodal involvement. Treatment options for these patients are usually limited due to the harmful cumulative radiation dose to nearby healthy organs when re-irradiated with conventional radiotherapy. This review aimed to explore the effectiveness of CyberKnife-based SRT in the management of re-irradiation for anatomical sites like the brain, lung, liver, spine, and prostate.

Technical aspects of the CyberKnife SRS/SABR technique

CyberKnife is an image-guided stereotactic dose delivery system designed for both focal delivery and SABR. Focal delivery refers to the use of several small beams to deliver a highly focused dose in several batches to a small target area. The system consists of a 6 MV linear accelerator mounted on a robotic arm coupled to a digital X-ray imaging system. The radiation doses are delivered using multiple beams to defined locations around the patient or nodal locations. CyberKnife can be used for both intracranial and extracranial treatments [2]. CyberKnife SRS is a promising treatment approach as it delivers effective and safe re-irradiation because of its ability to deliver a high biologically equivalent dose (BED) in a very precise manner while sparing healthy tissue, resulting in minimal toxicity [2]. It has been used to treat patients who have failed prior to RT, as well as those who are not surgical candidates.

Radiosurgery has become a powerful radiation technology in the past few decades, distinguished by its high degree of precision and quick radiation dose fall-off. The use of radiosurgery over the entire body is now possible owing to advancements in computation, and imaging technology, which was formerly restricted to complications within the brain, head, and neck. CyberKnife is one of the few devices capable of precisely targeting radiosurgery to tumors and other disorders throughout the body using cutting-edge imaging and robotic precision.

Dose Fractionation and Radiobiology (4R’s) of CyberKnife System

In 2011, Loo et al., who included SRS and SABR for the treatment of solid tumors, first introduced the concept of SABR [3]. Since tumor cells are directly ablated in response to high-dose radiation, SABR with a high dose per fraction and hypo-fractionated radiation produces convincing and satisfying therapeutic effects with low toxicity [4,5]. As a result, the problem of SABR insensitivity in tumors that are resistant to traditional EBRT may be resolved.

In SABR, high-dose radiation is administered in each fraction, and total doses are managed in two to five fractions over a very short time [6,7]. This results in more necroptosis than induction of apoptosis. Therefore, it is either very unlikely or impossible for tumor cells to be repaired. Accordingly, most tumor cells sustain fatal damage, resulting in cell death [7,8]. Additionally, in the context of SABR, a single high-dose ablation radiation treatment (for example, >20 Gy) results in complete blockage of the cell cycle at all stages. Since both sensitive and insensitive tumor cells are directly killed, redistribution of tumor cells is therefore not possible [9]. Under SABR, high-dose radiation ablates both oxygenated and hypoxic cells, effectively killing the tumor. Owing to the short duration of SABR, reoxygenation may be limited [10]. Furthermore, the SABR treatment plan is typically completed in two to five fractions within a week, leaving no time for the tumor cells to begin the repopulation process [11,12].

## Review

Methods

Literature Search Strategy

Contemporary articles were identified from the PubMed database accessed on May 01, 2023, for the time interval of 2006-2023. The present review is restricted to topics with sufficient literature, and the focus is primarily on re-irradiation using robotic radiotherapy at sites such as the head and neck, thorax, genitourinary (GU), pelvis, and spine. The keywords included “re-irradiation CyberKnife,” “reirradiation CyberKnife,” “re-irradiation robotic,” “reirradiation robotic,” “salvage CyberKnife,” “recurrent CyberKnife,” “repeat CyberKnife,” “retreatment robotic,” “re-treatment robotic,” “retreatment CyberKnife,” “re-treatment CyberKnife,” “recurrent CyberKnife,” and “recurrent robotic radiotherapy.” The inclusion criteria were as follows: (1) English language; (2) studies that treated recurrent disease using robotic (CyberKnife) SRT after the initial irradiation; and (3) studies with treatment efficacy details after re-irradiation. The exclusion criteria were as follows: (1) studies that reported fewer than four patients; (2) case reports; (3) studies reporting re-irradiation techniques other than the CyberKnife system; (4) studies with initial treatment without RT; and (5) studies with follow-up of less than six months.

The literature search resulted in a total of 51 studies with 3540 patients. The results were grouped by anatomical location of the re-irradiated region of the body, including the head and neck (n = 12; 605 patients), GU (n = 8; 719 patients), spine (n = 10; 873 patients), lung (n = 9; 912 patients), and pelvis (n = 12; 431 patients). The reported outcomes, toxicities, overall survival, prescription dose, follow-up, and local control rate were qualitatively reviewed in the present article.

Robotic SRS re-irradiation for brain/head & neck metastasis

Brain metastasis is a common adult intracranial malignancy that develops in 10-40% of patients with advanced-stage cancer during their treatment course [13]. Previously, whole-brain RT was the standard treatment for patients with multiple brain metastases. Surgical resection is the preferred salvage therapy for small recurrent or new primary head and neck tumors if the patient has previously received complete RT at the tumor site. Many patients cannot undergo surgery for technical or medical reasons. Re-irradiation of the brain may be the best example of the potential advantages of robotic SRT, which has a high rate of tumor control and minimal damage.

In total, 605 patients with 941 lesions were included in this review. The prescription doses for robotic SRT varied across the reviewed studies, ranging from 19 to 36 Gy/1-5 fractions. For re-irradiation cases with a median follow-up of 8.9-44 months, overall survival rates ranged from 3.6% to 61.7% for one year and 40.6% to 89.0% for two years, depending on the tumor type and location. Similarly, the local control rate ranged from 76.5% to 90.0% for one year and 41.2% to 73.0% for two years. The median survival was reported as 3-15.5 months. Nishizaki et al. presented the results of CyberKnife radiosurgery for 71 patients with 148 metastatic brain lesions [14]. At the median follow-up of 44 weeks, there were no permanent symptoms resulting from radiation necrosis. Overall, six-month and one-year survival rates were 74% and 47%, respectively, and the median survival time was 56 weeks. Local control was achieved in 83% of patients, and 25 patients developed 92 new metastases outside of the treated lesions with 22.4 months of median follow-up.

The survival of patients with brain metastases depends on many possible prognostic factors such as tumor volume, number of lesions per patient, marginal dose, number of fractions, control of metastases, primary lesions (lung or breast cancers vs. others), and extracranial metastases, as reported by Nishizaki et al. [14]. Berber et al. studied the clinical outcomes after CyberKnife radiosurgery re-irradiation for recurrent brain metastases. Seventy-seven patients with 254 lesions treated with SRS re-irradiation between January 2014 and December 2018 were analyzed [15]. Berber et al. observed an additional noteworthy clinical result, discovering that there was no correlation between regional recurrence and survival [15]. Sperduto et al. reported that controlled primary disease and extracranial metastases are important prognostic factors for survival [16]. Adachi et al. studied the feasibility of salvage re-irradiation with SRT for recurrent glioma using CyberKnife. Univariate analysis revealed that performance status at salvage re-irradiation was a significant predictor of progression-free survival [17]. Similarly, univariate analysis by Dogan et al. demonstrated that higher cumulative total radiotherapy dose, gross tumor volume, and recurrent time interval were prognostic factors for local failure-free survival [18]. Yamazaki et al. conducted a retrospective, multi-institutional analysis of 129 patients with previously irradiated cancer treated using robotic radiotherapy. The most frequent primary sites were the nasopharynx (43.4%) and oral cavity (24.8%). With a median follow-up duration of 10.6 months, the median overall survival was 14.4 months [19]. The data collected from various studies in brain metastases treatment are tabulated in Table 1.

**Table 1:** Clinical outcomes of CyberKnife stereotactic radiotherapy for head & neck and brain metastases from the selected studies

Study	No. of patients	No. of lesions	Median dose & number of fractions	Median follow-up in months	Local control rate	Overall survival	Overall toxicity	Median survival time
Nishizaki et al. (2006) [14]	71	148	20.7 Gy/1-3#	11 months	1 year - 83%	6 months - 74%; 12 months - 47%	At 44 weeks, no radiation necrosis	14 months
Hara et al. (2009) [20]	62	145	20 Gy/1#	10.5 months	1 year - 87%	6 months - 57%; 12 months - 37%	Radiation necrosis in 4 patients (6%)	8.3 months
Olson et al. (2012) [21]	27	-	20.5 Gy/1#	12 months	1 year - 76.5%	6 months - 25%; 12 months - 3.6%	No treatment-related toxicities	3 months
Greto et al. (2014) [22]	13	-	19.5 Gy/1-5#	9 months	6 months - 76.5%; 1 year - 76.5%	6 months - 75%; 12 months - 53%	Grade 2 toxicity	10 months
Tamari et al. (2015) [23]	67	109	24-30 Gy/1-3#	9.4 months	1-year - 83.3%	1 year - 54.8%; 3 years - 25.9%	-	13.1 months
de la Peña et al. (2018) [24]	49	152	20-26 Gy/1-5#	12 months	6 months - 96%; 1 year - 90%	15.5 months - 95%	No grade 3 toxicity	15.5 months
Balermpas et al. (2018) [25]	31	32	19 Gy/1-5#	12 months	1 year - 79.5%	1 year - 61.7%	Radio necrosis - 16.1%; Grade 3 - 12.9%	1 year
Adachi et al. (2019) [17]	35	48	9-36 Gy/1#	9 months	-	1 year - 41.3%	Grade 2 toxicity - 11.4%	9 months
Sayan et al. (2019) [26]	18	53	20 Gy/1-5#	10.4 months	-	12.6 months	Symptomatic radiation necrosis was not observed	12.6 months
Berber et al. (2021) [15]	77	254	24 Gy/3#	8.9 months	9 months - 73.4%,	9 months - 48%	Grade 3	8.9 months
Dogan et al. (2022) [18]	26	-	30 Gy/5#	44 months	3 years - 73.0%	3 years - 89.0%	Necrosis - 10.0%; trismus - 20.0%	-
Yamazaki et al. (2023) [19]	129	-		10.6 months	2 years - 41.2%	2 years - 40.6%	Grade 3 - 18.6%	14.4 months

Robotic SABR re-irradiation for hepatocellular carcinoma and liver metastases

The liver is one of the most common sites for the metastatic spread of cancer caused by colorectal, pancreatic, and breast cancers. Widely used surgical resection for the treatment of liver metastases has limitations due to a high rate of relapse and complicated procedures. Therefore, CyberKnife, an image-guided robotic radiosurgery system, has emerged as a promising treatment method with high efficacy and low toxicity for primary and metastatic liver tumors [27].

The liver is considered a radiosensitive organ, prone to radiation-induced hepatic diseases caused by RT, which can develop even after four to eight weeks of treatment. Stereotactic body radiation therapy (SBRT) was developed as a non-invasive way to administer local ablative therapy for restricted liver metastases, producing optimal local tumor control, a minimal dose to nearby healthy tissue, and perhaps decreased complication rates for radiation-induced liver disease (RILD). Therefore, various groups have demonstrated that SBRT using CyberKnife is feasible for liver metastases and have reported the clinical outcome of optimum doses and fractions for preventing RILDs.

The data collected from various studies on the treatment of liver and GU metastases are shown in Table 2. In total, 719 patients with 944 lesions were included in this review. The prescription doses for robotic SRT varied across the reviewed studies, ranging from 20 to 45 Gy/1-5 fractions. For re-irradiation cases with a median follow-up of 5.9-40.8 months, overall survival rates ranged from 18.0% to 94.0% for 12 months and 32.5% to 89.7% for two years, respectively. Similarly, the local control rate ranged from 60.4% to 94.4% for one year and 11.1% to 89.7% for two years. The median survival time was reported as 5.9-37.1 months. Lawrence et al. reported that patients irradiated by conventional fractionation with a mean dose of ~37 Gy developed radiation hepatitis [28]. This study also suggested that radiation hepatitis is infrequent at doses of approximately 30 Gy, and the risk increases significantly above 35 Gy. Benson et al. analyzed the dose constraints for SBRT. They recommended a mean dose of less than 13-18 Gy for three fractions and less than 15-20 Gy for six fractions of SBRT [29]. Son et al. emphasized that to minimize the likelihood of hepatic function decline during CyberKnife-based SBRT, the cumulative liver volume exposed to doses below 18 Gy should exceed 800 cm³ [30]. Hoyer et al. reported a 2% incidence of liver failure after 45 Gy in three fractions after SBRT [31]. Van der Pool et al. reported acute grade 3 liver toxicity in two of 20 patients after three fractions of 12.5 to 15 Gy per fraction [32]. Multiple studies have reported the application of CyberKnife SRT in the management of re-irradiation for hepatic tumors, pancreas, colorectal, and liver metastases [33-38]. Dewas et al. evaluated the use of SBRT for treating colorectal liver metastases in patients who could not undergo surgery or radiofrequency ablation. The SBRT was administered to 20 patients with one to three metastases, and the median size was 2.3 cm. Most patients experienced low-grade hepatic toxicity, with only two cases of grade 3 toxicity [33]. Stintzing et al. presented long-term survival data on the treatment of liver metastases using CyberKnife radiosurgery. The study focused on patients with limited liver metastases, employing curative intent. A total of 126 patients with 194 lesions were included, with a median follow-up of 30 months. The study concluded that single-session robotic radiosurgery demonstrates a high efficacy in controlling radiated lesions, offering curative potential for patients [38].

**Table 2:** Clinical outcomes of CyberKnife stereotactic radiotherapy for liver and genitourinary metastases from the selected studies

Study	No. of patients	No. of lesions	Median dose & number of fractions	Median follow-up in months	Local control rate	Overall survival	Overall toxicity	Median survival time
Dewas et al. (2011) [33]	42	62	45 Gy/3#	14.3 months	1 year - 90.0%; 2 years - 86.0%	1 year - 94%; 2 years - 48%	Grade 1 or grade 2	24 months
Dewas et al. (2012) [34]	120	153	45 Gy/3#	15 months	1 year - 84.0%; 2 years - 74.6%,	-	-	10.6 months
Lominska et al. (2012) [35]	28			5.9 months	6 months - 86.0%	1 year - 18%	Grade 3 < 1%	5.9 months
Yuan et al. (2014) [27]	4	80	42 Gy/3#	12 months	1 year - 94.4%; 2 years - 89.7%	2 years - 72.2%	No grade 3 or higher	37.5 months
Sutera et al. (2018) [36]	38	-	24.5 Gy/1-3#	24.4 months	2 years - 82.0%	26.6 months - 95.0%; 2 years - 53%	Grade 2 - 18.4%; Grade 3 - 10.5%	26.6 months
Vernaleone et al. (2019) [37]	38	66	37.5 Gy/3#	11.8 months	6 months - 64.2%; 12 months - 60.4%	1 year - 67.3%; 2 years - 44.1%	No acute grade 3	20.1 months
Stintzing et al. (2019) [38]	126	194	20-45 Gy	40.8 months	1 year - 94.1%	3 years - 47.7%; 5 years - 32.5%		35.2 months
Kibe et al. (2020) [39]	323	389	35-40 Gy/5#	37.1 months	3 years - 11.1%	3 years - 66.1%	No grade 3 was observed	37.1 months

Robotic SBRT re-irradiation for spine metastases

In the treatment of spinal metastases, conventional radiotherapy has limitations due to the relatively low tolerance of the spinal cord to radiation, as conventional external beam radiotherapy lacks the precision to deliver large single-fraction doses of radiation to the spine near radiosensitive structures, such as the spinal cord. Hence, researchers have investigated various ways to improve spinal irradiation in an effort to improve local control and reduce radiation toxicity. SBRT, a new paradigm for spinal metastases, delivers a high dose of radiation in a single fraction to a well-defined target accurately. Therefore, SBRT is considered to be very effective for spinal radiosurgery. CyberKnife spinal radiosurgery treatment consists of precise robotic stereotactic treatment based on tumor geometry, proximity to the spinal cord, and location. Multiple studies have confirmed the efficacy of SBRT, with early symptomatic improvement in 86-96% of treated patients [40,41].

Table 3 displays the information gathered from the articles evaluating robotic radiotherapy in the management of spinal metastases in the last two decades. In total, 873 patients with approximately 870 lesions were included in this review. The prescription doses for robotic SRT varied across the reviewed studies, ranging from 20 to 38 Gy/1-5 fractions. For re-irradiation cases with a median follow-up of six to 34 months, overall survival rates ranged from 48.0% to 80.0% for 12 months and 45.0% to 57.0% for two years, respectively. Similarly, the local control rate ranged from 76.0% to 90.0% for one year and 69.0% to 73.0% for two years and therefore seems to be promising. The median survival time was reported to be 11-29 months. A relatively low incidence of myelopathy was seen overall. In the study by Choi et al., one out of 42 patients (incidence: 2%) experienced grade 4 spinal cord damage [42]. A cohort of 500 cases of spinal metastases underwent radiosurgery. Locations of the lesions included 73 cervical, 212 thoracic, 112 lumbar, and 103 sacral. The maximum intra-tumoral dose ranged from 12.5 to 25 Gy (mean = 20). Long-term pain improvement occurred in 290 of 336 cases [40]. Furuya et al. presented the dosimetric analysis of spine SBRT re-irradiation using three different beam delivery techniques: static-field intensity modulated radiation therapy (SIMRT), volumetric-modulated arc therapy (VMAT), and CyberKnife in terms of its suitability for spine SBRT [43]. It was reported that irrespective of the technique employed for administration, the quality of the treatment plan is more significantly influenced by the spinal cord's dose tolerance than by the tumor volume [43]. Emma et al. reported a retrospective series of 49 spinal segments treated with CyberKnife SBRT and noticed an 18% incidence of pseud progression at a median of five months (range = 3-9 months) from treatment completion [44]. In light of these data, it may be concluded that SBRT can be used as a safe and effective alternative to traditional radiotherapy in cases of re-treatment when the spinal dosage tolerance cannot be met. However, more research is necessary to fully understand spinal recovery and spinal cord tolerance to inhomogeneous SBRT dose distributions.

**Table 3:** Clinical outcomes of CyberKnife stereotactic radiotherapy for spinal metastases from the selected studies

Study	No. of patients	No. of lesions	Median dose & fractions	Median follow-up in months	Local control rate	Overall survival	Overall toxicity	Median survival time
Gerszten et al. (2007) [40]	500	500	20 Gy/1#	-	90.0%	-	No acute radiation toxicity	-
Sahgal et al. (2009) [45]	39	60	24 Gy/3#	9 months	1 year - 85.0%; 2 years - 69.0%	2 years - 45.0%	No toxicity reported	21 months
Choi et al. (2010) [42]	42	51	20 Gy/1-5#	7 months	1 year - 81.0%	6 months - 73.0%; 1 year - 68.0%	Grade 4 neurotoxicity - 2%	27 months
Mahadevan et al. (2011) [46]	60	81	24 Gy/3#	12 months	1 year - 90.0%	1 year - 80% ; 2 years - 60.7%,	No significant toxicity	11 months
Garg et al. (2011) [47]	59	63	27 Gy/3# or 30 Gy/5#	17.6 months	1 year - 76.0%	1 year - 76%	Grade 1 and grade 2 observed	-
Chang et al. (2012) [48]	54	59	20.6 Gy/1#	17.3 months	1 year - 81.0%	-	No case of radiation myelopathy	29 months
Gill et al. (2012) [41]	20	-	30-35 Gy/5#	34 months	1 year - 80.0%; 2 years - 73.0%	1 year - 80.0%; 2 years - 57.0%,	No spinal cord toxicity	-
Sohn et al. (2014) [49]	13	-	38 Gy/4#	6 months	1 year - 85.0%	-	Grades 1 and 2	15 months
Thibault et al. (2015) [50]	40	56	30 Gy/4#	6.8 months	1 year - 81.0%	1 year - 48.0%	No cases of myelopathy	10 months
Hu et al. (2020) [51]	46	-	35 Gy/5#	16 months	1 year - 90.0%	1 year - 76.0%	No radiation-induced myelopathy	18 months

Robotic SBRT re-irradiation for lung metastases

Treatment of lung metastases with EBRT has resulted in loco-regional relapse in up to 50% of patients. Later, the development of SBRT has brought about a paradigm shift in lung metastasis treatment, since it can produce high rates of tumor control with very low toxicity. SBRT for primary lung cancers and lung metastases has shown excellent clinical results and has become an established standard treatment option.

In total, 912 patients with approximately 1020 lesions were included in this review. The prescription doses for robotic SBRT varied across the reviewed studies, ranging from 6 to 54 Gy/3-8 fractions. For re-irradiation cases with a median follow-up of 9-24 months, overall survival rates ranged from 52.3% to 88.4% for 12 months and 42.0% to 95.0% for two years, respectively. Similarly, the local control rate ranged from 75.0% to 98.8% for one year and 50.0% to 95.0% for two years. The median survival time was reported to be three to 38 months. Table 4 provides information from the articles on treating lung metastases. Patients with primary lung cancer have a 10% probability of developing a secondary lung tumor within five years after treatment, requiring re-irradiation. Therefore, the demand for repeated lung irradiation is high. However, it carries a risk of radiation-induced lung injury (radiation pneumonitis and radiation fibrosis) [52,53]. There are still insufficient clinical data to determine the optimal tumor selection parameters, such as dose, fractionation schedules, local control, and potential survival rates. Therefore, this review presents various parameters of lung metastasis treatment as tabulated in Table 4. Biswas et al. described the experience and outcomes of treating primary and secondary lung cancers using CyberKnife SBRT. Results showed a high overall response rate of 98.8%, with a complete response (CR) observed in 73.5% of cases. Furthermore, the study suggests that the use of tight planning target volume (PTV) margins and adequate dosimetric coverage contributed to reasonable treatment outcomes [54]. Wang et al. aimed to assess the trends and impact of postoperative radiation therapy (PORT) on overall survival in patients with incompletely resected stage II-III non-small cell lung cancer (NSCLC). The study concluded that PORT is linked to enhanced overall survival in patients with incompletely resected stage II-III N0-N2 NSCLC, even though its utilization is decreasing in more recent years [55].

**Table 4:** Clinical outcomes of CyberKnife stereotactic radiotherapy for lung metastases from the selected studies

Study	No. of patients	No. of lesions	Median dose & fractions	Median follow-up in months	Local control rate	Overall survival	Overall toxicity	Median survival time
Biswas et al. (2012) [54]	79	83	50 Gy/3- 5#	13.1 months	1 year - 98.8%	76%	Grade 2 - 0.1%; grade 3 - 0.1%	13 months
Meijneke et al. (2013) [52]	20	-	50-60 Gy/3-5#	12 months	1 year - 75.0%; 2 years - 50.0%	1 year - 67%; 2 years - 33%	No grade 3 - 5 toxicity	3-8 months
Wang et al. (2015) [55]	95	134	30-60 Gy/1-5#	17 months	1 year - 97.6%; 2 years - 90.6%; 3 years - 87.0%	2 years - 61.3%	No higher toxicity	38 months
Patel et al. (2015) [53]	26	29	25 Gy/5#	18 months	1 year - 78.6%; 2 years - 65.5%	1 year - 52.3%; 2 years - 37.0%	Grade 1 or 2 toxicity	14 months
Ceylan et al. (2017) [56]	28	34	6-30 Gy/3-8#	9 months	2 years - 67.0%	1 year - 71.0%; 2 years - 42.0%	No grade 3 or higher	21 months
Viani et al. (2020) [57]	595	625	37 Gy/2#	-	2 years - 95.0%	2 years - 95%	Grade 3 - 1.5%	-
Ricco et al. (2020) [58]	44	88	50-54 Gy/3-4#	24 months	6 years - 82.7%	3 years - 34.1%	Grade 3 - 4.5%	24 months
Kinj et al. (2021) [59]	5	-	60 Gy/8#	11.1 months	-	-	No grade 3	-
Shou et al. (2022) [60]	20	27	60 Gy/5#	18.0 months	1 year - 95.2%	1 year - 88.4%; 2 years - 49.7%	Grade 2 or higher toxicity	23 months

Robotic SRT re-irradiation for pelvis/prostate metastases

RT is often the first-line treatment for pelvic cancers such as rectal adenocarcinoma, prostate cancer, and gynecologic tumors. In total, 431 patients with approximately 62 lesions were included in this review. The prescription doses for robotic SRT varied across the studies reviewed ranging from 8 to 36.25 Gy/1-5 fractions. For re-irradiation cases with a median follow-up of 10.6-38.6 months, overall survival rates ranged from 46.0% to 95.0% for 12 months and 37.0% to 89.2% for two years, respectively. Similarly, the local control rate ranged from 51.4% to 92.0% for one year and 50.0% to 95.0% for two years. The median survival was reported as 8.3-43 months. Data compiled from the articles on the treatment of spinal metastases are presented in Table 5. Dewas et al. published a retrospective study of 16 patients re-irradiated with CyberKnife for lateral pelvic recurrence [34]. The patients included those with primary anal canal cancer (six patients), rectal cancer (four patients), cervical cancer (four patients), endometrial cancer (one patient), and treatment for a recurrence of bladder cancer (one patient). The patient had previously been treated with a median radiation dose of 45 Gy (range: 20-96 Gy), and the median interval between the first radiotherapy cycle and retreatment was 5.1 months. In the second course of SBRT, 36 Gy was administered in six doses over three weeks. The median follow-up was 10.6 months (range = 1.9-20.5 months). The authors reported a one-year local control rate of 51.4%, median disease-free survival (DFS) of 8.3 months, and one-year actuarial survival rate of 46%. Patients with adenocarcinoma tended to have better local control than those with squamous cell carcinoma (p = 0.09). Acute and late toxicities were classified as grade ≤ 2 [57]. The purpose of Abusaris et al.'s study was to explore the outcome, cumulative dose in tumors and organs at risk, and toxicity after extracranial stereotactic re-irradiation [61]. Defoe et al. aimed to assess the effectiveness and safety of CyberKnife SBRT for managing recurrent presacral rectal cancer. Most patients had prior radiotherapy, and the median tumor volume was 52.5 cc. The study reported CyberKnife SBRT to be an effective and well-tolerated treatment that offers palliation of pain and contributes to positive local control and overall survival rates [62]. Similarly, multiple studies have reported the use of CyberKnife stereotactic re-irradiation for prostate cancer [63-68]. Fuller et al. aimed to assess the efficacy and safety of high-dose-rate-like SBRT retreatment for biopsy-proven local persistence of recurrent prostate cancer after prior RT. Results showed that median pre-SBRT prostate-specific antigen (PSA) levels decreased significantly after retreatment. The five-year biochemical DFS rate was 60%, with favorable rates of local, distant, and salvage androgen deprivation therapy-free survival. Further, the approach demonstrated low GU and GI toxicity, providing a potential treatment option for this challenging patient group [68].

**Table 5:** Clinical outcomes of CyberKnife stereotactic radiotherapy for pelvis metastases from the selected studies

Study	No. of patients	No. of lesions	Median dose & fractions	Median follow-up in months	Local control rate	Overall survival	Overall toxicity	Median survival time
Defoe et al. (2011) [62]	14	-	36 Gy/3# or 12-18Gy/1#	16.5 months	1 year - 90.0%; 2 years - 68.2%	1 year - 90.0%; 2 years - 78.8%	No grade 3 or grade 4 toxicity	-
Dewas et al. (2011) [33]	16	-	36 Gy/6#	10.6 months	1 year - 51.4%	1 year - 46.0%	Grade 1 - 6.25%; grade 2 - 12.5%	8.3 months
Abusaris et al. (2012) [61]	27	-	8-20 Gy/2-3#	15 months	1 year - 53.0%; 2 years - 40.0%	1 year - 52.0%; 2 years - 37.0%	Grade 1 - 15.0%; grade 2 - 7.0%	14 months
Dagoglu et al. (2015) [63]	18	27	25 Gy/5#	-	89.0%	-	Grade 4 - 1 pt; grade 3 - 2 pts	43 months
Leroy et al. (2017) [64]	23		36 Gy/6#	22.6 months	2 years - 76.0%	1 year - 100.0%	Grade 2 - 78.2%; grade 3 - 8.7%	24 months
Loi et al. (2018) [65]	50		30 Gy/5#	21.3 months	1 year - 92.0%	1 year - 82%	Grade 2 - 20%; grade 3 - 2%	12 months
Jereczek-Fossa et al. (2018) [66]	64		30 Gy/5#	26.1 months	2 years - 75.0%	1 year - 92%	Grade 2 - 25%; grade 3 - 1.5%	24 months
Scher et al. (2019) [67]	42		36 Gy/6#	21 months	11 months - 100.0%	-	Grade 2 - 21%; grade 3 - 2%	11 months
Fuller et al. (2020) [68]	50		34 Gy/5#	44 months	2 years - 76.0%; 5 years - 60.0%		Grade 2 - 2.2%; grade 3 - 8%	2 years
Smith et al. (2020) [69]	30	35	30 Gy/5#	24.5 months	1 year - 84.9%; 2 years - 69.0%	1 year - 95.0%	Grade 3 - 1 patient	28.3 months
Miszczyk et al. (2023) [70]	56	-	36.25 Gy/5#	38.6 months	2 years - 87.6%; 5 years - 47.9%	2 years - 89.2%; 5 years - 48.5%	Grade 3 - 32.1%	-
Allali et al. (2023) [71]	41	-	-	35 months	2 years - 93.6%	2 years - 72.9%	Grade 3 - 2 pts	2 years

Cost-effectiveness of robotic radiotherapy

As SBRT is increasingly used in clinical practice, it is imperative to assess its cost-effectiveness and efficacy. SBRT with image guidance, high-precision dose delivery, better anatomical and biological imaging for a more accurate target definition, and the possibility of dose verification during treatment with dose-adaptive radiotherapy will improve tumor control more likely. Such major technological advances come at a high cost, and there are many concerns regarding their value. However, the increased cost of equipment and resources associated with state-of-the-art radiation oncology techniques can be partially mitigated by reducing the number of treatment cycles, in addition to better tumor control and less toxicity. A course of treatment reduces the indirect costs of cancer treatment, such as lost time and economic productivity resulting from treatment-related and cancer-related illnesses and deaths. Treatment outcomes for SRS and SBRT are usually superior or comparable and cost-effective relative to alternative techniques [72].

Discussion

The evidence for retreatment after primary radiotherapy is low. Due to their retrospective nature, study materials vary widely with respect to pre-treatment, dose differences, and primary radiotherapy dose. Additional treatments, such as surgery and chemotherapy combined with radiation treatment of recurrence, vary widely within and between cohorts, with cohorts differing in age, sex, and comorbidities [73]. The advantage of SBRT is the sparing of normal tissue. This is the most important factor in the recurrence scenarios. SBRT, however, is most commonly administered in extremely low fractions. When the concept of SBRT was invented, technology and resources dictated the use of lower doses. Currently, SBRT is still performed using hypofractionation techniques, but it is now primarily explained by the belief in biological benefits. However, if there is a high risk of sequelae or complications due to radiation-sensitive organs in the vicinity of the target, the use of other fractions makes sense. This is often the case for SBRT in re-irradiation scenarios. Tissue regeneration from occult damage during first-line radiation has been thoroughly reported for various organs at risk. However, after re-irradiation, no experimental animal experiments have been conducted. The studies examined also have a number of other limitations that need to be acknowledged. The extent of the overlap between the three irradiation volumes was calculated using various techniques. To determine the true cumulative dose, such estimations require sophisticated image registration techniques to consider inter- and intra-fraction mobility as well as long-term anatomical changes. Most studies had minimal follow-up and/or median survival, making it difficult to determine long-term toxicity [74].

The reserve capacity is frequently significant in parallel-organized tissues such as the peripheral lung and liver. The reserve capacity may compensate for the loss of organ function, regardless of whether the primary and relapse goals overlap. According to imaging studies, liver tissue that has received low and intermediate doses may recover to some extent in the months immediately following the main SBRT [75]. However, it is unclear whether comparable mechanisms occur in other organs. SBRT re-irradiation therapy can be used in many cases where cancer recurs locally in the body after primary RT. The most significant limitation in the use of SBRT re-irradiation is the lack of knowledge regarding the efficacy and tolerability of re-irradiation. The current review provides sufficient details on the clinical efficacy of robotic radiotherapy in the management of re-irradiation scenarios for different anatomical sites. Furthermore, reporting methods of image registration between repeated treatments and dose accumulation to individual organs at risk should be recommended.

This research contributes to the existing literature by specifically addressing the application of robotic SRT for re-irradiation. It likely builds upon previous studies on both re-irradiation techniques and the use of robotic SRT, providing valuable insights into a potentially novel treatment approach. The ability of CyberKnife-based SRT to deliver precise doses while sparing healthy tissue and the reported outcomes from various anatomical sites all support the claim that CyberKnife-based re-irradiation is an effective treatment approach. The study's emphasis on minimal toxicity and tumor control aligns with the evidence provided.

Limitations

In this comprehensive review, articles published prior to 2006 were deliberately omitted. Solely the PubMed database was employed as the search platform. The evidence reported in this review is limited due to the fact that no controlled studies have been published, and studies included are highly heterogeneous in-patient populations and interventions. Additionally, outcomes such as local control, progression-free survival, and pain response are reported in a variety of ways.

## Conclusions

SBRT has emerged as a promising technique for the re-irradiation of metastases in various organs, owing to its excellent results in terms of tolerance and high local control rates. Robotic-based SRT in patients undergoing re-irradiation has been shown to be an effective treatment option, with excellent local control rates and low toxicity across various anatomical sites. The technical aspects of the CyberKnife system allow for highly conformal radiation delivery and real-time tracking of tumor motion, whereas the radiobiological effects of high-dose radiation therapy result in tumor cell killing and minimal normal tissue toxicity. Further research is needed to optimize treatment protocols and identify the patient population that will benefit the most from this approach. However, current evidence suggests that CyberKnife-based robotic SRT is a promising treatment option for patients undergoing re-irradiation. This review has presented the significance of SBRT administered using CyberKnife techniques, dose and fractionation schemes, and other limitations. Currently, SBRT is a widely used treatment option for various metastases, requiring experienced multidisciplinary teams to further optimize the treatment parameters to control toxicity and radiation-induced diseases.
